# COVID-19-Associated Encephalitis: Two Case Reports

**DOI:** 10.7759/cureus.23243

**Published:** 2022-03-17

**Authors:** Leandro M Marques, Sofia R Marques, Octávia Costa, Eduardo Freitas, Álvaro Machado

**Affiliations:** 1 Neurology, Hospital de Braga, Braga, PRT

**Keywords:** viral meningitis, sars-cov-2-associated encephalitis, covid-19 encephalitis, acute encephalitis, sars-cov-2, covid 19

## Abstract

The infection with SARS-CoV-2 is primarily associated with respiratory symptoms. Since its appearance, several neurological symptoms have been reported, most commonly headache and anosmia, as well as less frequent complications such as COVID-19-associated encephalitis and meningitis. In this case report, we describe two patients, who were 49- and 50-year-old infected with SARS-CoV-2, who presented to the emergency department with altered mental status and behavioral changes. A diagnosis of acute meningoencephalitis associated with COVID-19 was considered, and both patients had a good response to corticosteroid treatment.

## Introduction

Coronavirus disease 19 (COVID-19) is a respiratory disease caused by the severe acute respiratory syndrome coronavirus 2 (SARS-CoV-2). The spectrum of clinical manifestations is wide, ranging from asymptomatic/subclinical infection to acute respiratory distress syndrome and respiratory failure that requires intubation and intensive care [1].

The COVID-19 also involves other systems, including the nervous system. The neurological involvement of SARS-CoV-2 is variable, resulting from central and peripheral nervous system manifestations, mostly headache, myalgias and impaired consciousness, but also encephalopathy, skeletal muscle injury, acute polyradiculoneuropathys, acute cerebrovascular events, meningitis, encephalitis and encephalomyelitis. The proportion of patients presenting with neurological complications is higher in severe cases of SARS-CoV-2 infections [2,3].

The precise mechanism by which SARS-CoV-2 causes meningitis and encephalitis is not well described, although some authors reported detection of SARS-CoV-2 RNA in cerebrospinal fluid (CSF) and brain tissue [4,5].

COVID-19-associated encephalitis is not widely reported, with only a few dozen reports published, and the specific clinical characteristics are not well detailed. We describe two cases of COVID-19-associated encephalitis and their outcome.

## Case presentation

Patient 1

A 49-year-old female with no past medical history presented to the emergency department with altered mental status since that day. She was seen in her primary care provider's clinic six days before with symptoms of fever and myalgias, and the diagnosis of COVID-19 was made after a positive polymerase chain reaction (PCR) assay for SARS-CoV-2 in nasopharyngeal swab (variant analysis was not performed).

**Figure 1:**
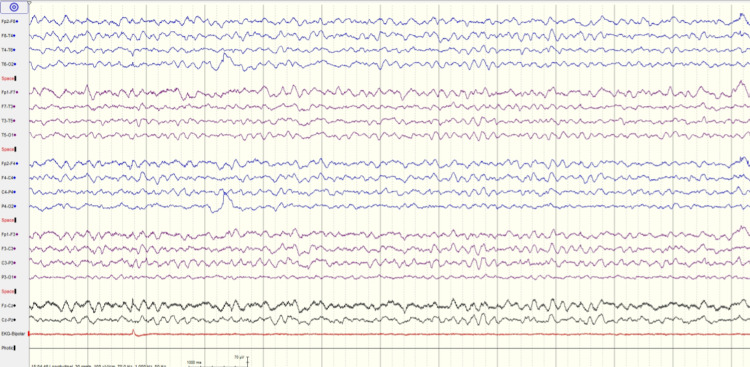
EEG recording of patient 1 showing the posterior background of 6 Hz, moderate diffuse encephalopathy without epileptiform activity

Initial vital signs were normal; O_2_ saturation was 98% breathing room air. On physical examination, the patient was lethargic, not oriented to time and place, could not follow commands. Neurological examination revealed neck rigidity, but not other focal neurological deficits. Blood investigations, with viral serology for herpes simplex virus (HSV) 1-2, human immunodeficiency virus (HIV) and varicella-zoster (VZV) were within normal range except for elevated lactate dehydrogenase and d-dimers. Septic workup, including blood and urine cultures, was negative. Her urine toxicology was negative. Chest x-ray and computed tomography (CT) scan were normal. CT scan of the head and CT angiogram of the neck and brain were normal. She underwent a lumbar puncture and was started empirically on antibiotic and antiviral (ceftriaxone and acyclovir) for meningitis treatment. Lumbar puncture showed an opening pressure of 17 cm of H_2_O; her CSF results are shown in Table 1. Magnetic resonance imaging (MRI) of the brain did not show any pathological changes. Electroencephalography (EEG) was performed, which showed moderate encephalopathy, without epileptiform activity (Figure 1).

**Table 1:** Cerebrospinal fluid analysis of patient 1. * 90.9% mononuclear leukocytes and 9.1% polymorphonuclear leukocytes

Cerebrospinal fluid	Values	Reference range
Color	Colorless	Colorless
Opening pressure (cmH2O)	17	5-20
White blood cell count (mm3) *	11	0-5
Red blood cells (mm3)	0	0
Glucose (mg/dl)	59	40-70
Protein (mg/dl)	82	15-45
Gram stain	Negative	Negative
Cryptococcal antigen	Negative	Negative
SARS-CoV-2 PCR	Negative	Negative
Herpes simplex virus 1 and 2 PCR	Negative	Negative
Varicella-zoster vírus PCR	Negative	Negative
Cytomegalovirus PCR	Negative	Negative

A diagnosis of meningitis/encephalitis associated with the SARS-CoV-2 infection was made. After the results of CSF analysis, antibiotic and antiviral medications were stopped. Intravenous dexamethasone (6 mg once per day) was added on the second day of symptoms. Her neurological symptoms started improving after 12 hours. After 72 hours of admission, her neurological examination was normal. She had amnesia for all the events since the beginning of the symptoms. She was discharged home on day 8. Two months post-discharge, she was doing well with no neurological signs and symptoms.

Patient 2

A 50-year-old female with a past medical history of depression presented to the emergency department with behavioral changes started a few hours before. She was seen in her primary care provider's clinic eight days before with symptoms of headaches and asthenia, and the diagnosis of COVID-19 was made after a PCR assay for SARS-CoV-2 in nasopharyngeal swab (variant analysis was not performed).

**Figure 2:**
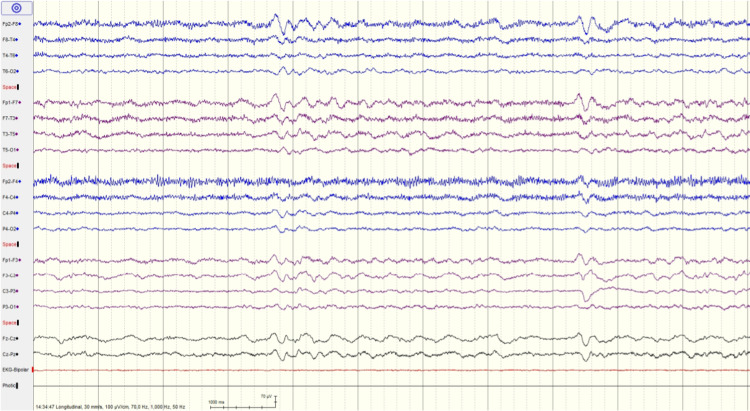
EEG recording of patient 2 showing posterior background of 7 Hz, mild diffuse encephalopathy without epileptiform activity

Initial vital signs were normal; O_2_ saturation was 100% breathing room air. On physical examination, the patient was restless, sometimes physically aggressive did not follow commands and was in mutism. The neurological examination, besides the mutism, did not reveal other focal neurological deficits. Blood investigations, with viral serology for HSV 1-2, HIV and VZV, were within normal range. Septic workup, including blood and urine cultures, was negative. Her urine toxicology was negative. Chest x-ray and CT scan were normal. CT scan of the head and CT angiogram of the neck and brain were normal. She underwent a lumbar puncture and was started empirically on antibiotic and antiviral (ceftriaxone and acyclovir) for meningitis treatment. Lumbar puncture showed an opening pressure of 13 cm of H_2_O; her CSF results are shown in Table 2. MRI of the brain did not show any pathological changes. An EEG was performed, that showed mild encephalopathy, without epileptiform activity (Figure 2).

**Table 2:** Cerebrospinal fluid analysis of patient 2.

Cerebrospinal fluid	Values	Reference range
Color	Colorless	Colorless
Opening pressure (cmH2O)	13	5-20
White blood cell count (mm3) *	1	0-5
Red blood cells (mm3)	800	0
Glucose (mg/dl)	93	40-70
Protein (mg/dl)	16	15-45
Gram stain	Negative	Negative
Cryptococcal antigen	Negative	Negative
SARS-CoV-2 PCR	Negative	Negative
Herpes simplex virus 1 and 2 PCR	Negative	Negative
Varicella-zoster vírus PCR	Negative	Negative
Cytomegalovirus PCR	Negative	Negative

A diagnosis of encephalitis associated with the SARS-CoV-2 infection was made. After the results of CSF analysis, antibiotic and antiviral medications were stopped. Intravenous dexamethasone (4 mg once per day) was added on the second day of symptoms. Her neurological symptoms started improving after 24 hours. After 96 hours of admission, her mental status and neurological examination were normal. She had amnesia for all the events since the beginning of the symptoms. She was discharged home on day eight. Three months post-discharge, she was doing well with no neurological signs and symptoms.

## Discussion

The pathophysiological mechanism of acute encephalitis in COVID-19 is not well defined. There are two main hypotheses: a direct cytopathic effect of the infection of the brain tissue (the angiotensin-converting enzyme 2 receptors that the virus uses for attachment, margination, and internalization in the lung, are also expressed in the central nervous system; viral antigens were detected in CSF and brain samples), and an autoimmune/immune-mediated cause (the general hyperinflammatory state releases cytokines and chemokines that impairs the blood-brain barrier permeability and activate neuro-inflammatory cascades) [6,7]. In our cases, we presume that the immune-mediated was the causative agent, because of the excellent response to IV steroids.

Acute encephalitis diagnosis is based on clinical presentation, CSF analysis, and brain imaging. However, there are no diagnostic criteria defined in COVID-19-induced encephalitis. The initial symptoms vary, although changes in consciousness, seizures and meningeal irritation signs were among the most frequently reported. The CSF analysis may exhibit increased protein level, increased white blood cell level and positive PCR results for SARS-CoV-2. Neuroimaging mainly shows abnormal findings of brain damage. EEG may show a slowing of background, diffuse slow-wave, or focal epileptic wave [8]. But none of this is mandatory, so the diagnosis of COVID-19-related encephalitis can be remarkably challenging. In our cases, the PCR assay for SARS-CoV-2 was negative, not allowing to do a definitive diagnosis of viral encephalitis. A case series involving CSF analysis data from 32 patients with SARS-CoV-2-associated encephalitis/meningitis reported that the PCR assay for SARS-CoV-2 in the CSF was negative in 87.5% of the patients [8] and in another case series with 40 patients CSF analysis PCR assay for SARS-CoV-2 was negative in 90% of the patients [7].

The treatment of COVID-19-related encephalitis is mostly supportive. There are reports of different treatments (IV steroids, IV immunoglobulin, plasmapheresis, immunomodulators) tried in various cases, with a good outcome in the majority of patients, but there are several case reports with a bad outcome, with case series reporting mortality rates as high as 10%, demonstrating the possible severity of the disease [8-10]. In our cases, the acute encephalitis induced by COVID-19 had a benign course, the patients improved quickly after IV steroids treatment and returned to their baseline state of health.

Our cases are of particular interest because they demonstrate that there is a clinical variety of symptoms caused by the COVID-19-related encephalitis and that is possible that the disease manifests without any specific findings in the blood work, CSF, EEG and MRI, even when there is no evidence of respiratory infection.

## Conclusions

Although SARS-CoV-2 is mainly a respiratory pathogen, neurological manifestations are not rare. A suspicion for COVID-19-associated encephalitis should be high among patients with SARS-CoV-2 infection and presenting acute neuropsychiatric manifestations. However, it has not been established if there are specific clinical findings. It is crucial to collect the most data about this disease to try to define diagnostic criteria and the most adequate treatment.
